# Identification of glycolysis-related gene signatures for prognosis and therapeutic targeting in idiopathic pulmonary fibrosis

**DOI:** 10.3389/fphar.2025.1486357

**Published:** 2025-02-28

**Authors:** Han Gao, Zhongyi Sun, Xingxing Hu, Weiwei Song, Yuan Liu, Menglin Zou, Minghui Zhu, Zhenshun Cheng

**Affiliations:** ^1^ Department of Respiratory and Critical Care Medicine, Zhongnan Hospital of Wuhan University, Wuhan, China; ^2^ Department of Critical Care Medicine, Zhongnan Hospital of Wuhan University, Wuhan, Hubei, China; ^3^ Fourth Ward of Medical Care Center, Hainan General Hospital, Hainan Affiliated Hospital of Hainan Medical University, Haikou, China; ^4^ Wuhan Research Center for Infectious Diseases and Cancer, Chinese Academy of Medical Sciences, Wuhan, China; ^5^ Hubei Engineering Center for Infectious Disease Prevention, Control and Treatment, Wuhan, China

**Keywords:** IPF, glycolysis, immune microenvironment, pharmacological strategies, model

## Abstract

**Background:**

Glycolysis plays a crucial role in fibrosis, but the specific genes involved in glycolysis in idiopathic pulmonary fibrosis (IPF) are not well understood.

**Methods:**

Three IPF gene expression datasets were obtained from the Gene Expression Omnibus (GEO), while glycolysis-related genes were retrieved from the Molecular Signatures Database (MsigDB). Differentially expressed glycolysis-related genes (DEGRGs) were identified using the “limma” R package. Diagnostic glycolysis-related genes (GRGs) were selected through least absolute shrinkage and selection operator (LASSO) regression regression and support vector machine-recursive feature elimination (SVM-RFE). A prognostic signature was developed using LASSO regression, and time-dependent receiver operating characteristic (ROC) curves were generated to evaluate predictive performance. Single-cell RNA sequencing (scRNA-seq) data were analyzed to examine GRG expression across various cell types. Immune infiltration analysis, Gene Set Enrichment Analysis (GSEA), and Gene Set Variation Analysis (GSVA) were performed to elucidate potential molecular mechanisms. A bleomycin (BLM)-induced pulmonary fibrosis mouse model was used for experimental validation via reverse transcription-quantitative polymerase chain reaction (RT-qPCR).

**Results:**

14 GRGs (*VCAN, MERTK, FBP2, TPBG, SDC1, AURKA, ARTN, PGP, PLOD2, PKLR, PFKM, DEPDC1, AGRN, CXCR4*) were identified as diagnostic markers for IPF, with seven (*ARTN, AURKA, DEPDC1, FBP2, MERTK, PFKM, SDC1*) forming a prognostic model demonstrating predictive power (AUC: 0.831–0.793). scRNA-seq revealed cell-type-specific GRG expression, particularly in macrophages and fibroblasts. Immune infiltration analysis linked GRGs to imbalanced immune responses. Experimental validation in a bleomycin-induced fibrosis model confirmed the upregulation of GRGs (such as AURKA, CXCR4). Drug prediction identified inhibitors (such as Tozasertib for AURKA, Plerixafor for CXCR4) as potential therapeutic agents.

**Conclusion:**

This study identifies GRGs as potential prognostic biomarkers for IPF and highlights their role in modulating immune responses within the fibrotic lung microenvironment. Notably, *AURKA, MERTK*, and *CXCR4* were associated with pathways linked to fibrosis progression and represent potential therapeutic targets. Our findings provide insights into metabolic reprogramming in IPF and suggest that targeting glycolysis-related pathways may offer novel pharmacological strategies for antifibrotic therapy.

## Introduction

Idiopathic pulmonary fibrosis (IPF) is a chronic, progressive pulmonary fibrosis characterized by unexplained fibrosis and scarring of lung tissue, This condition is marked by the abnormal proliferation and activation of fibroblasts, which leads to extensive lung remodeling and ultimately results in impaired lung function (Podolanczuk et al., 2023; Moss et al., 2022; Wang et al., 2022). IPF relies primarily on antifibrotic treatments and various palliative and supportive care. Lung transplantation remains the only effective option for extending survival, but its accessibility and success rates are constrained by donor organ availability (Glassberg, 2019; Maher et al., 2021; Shah Gupta et al., 2023). Despite substantial research into the pathophysiological mechanisms underlying IPF, the precise etiology of this debilitating disease remains elusive, and effective therapeutic interventions remain scarce.

Glycolysis, the primary pathway of glucose metabolism, plays a central role in cellular energy production and is intricately linked to various cellular processes such as cell proliferation, inflammatory responses, and apoptosis (Bose and Le, 2018; Koppenol et al., 2011). The process of glycolysis is not only critical for maintaining cellular homeostasis but also for fueling the metabolic demands of activated fibroblasts and other cells in fibrotic tissues. Recent studies have underscored the importance of glycolysis in the development and progression of a wide range of diseases, including tumors, chronic inflammatory conditions, and fibrotic diseases such as IPF (Mulukutla et al., 2016; Vander Heiden et al., 2009; Lunt and Vander Heiden, 2011). In IPF, glycolysis is thought to be upregulated, providing the energy required for the activated fibroblasts that contribute to lung fibrosis. However, despite growing evidence of its involvement, the precise molecular mechanisms linking glycolysis to IPF pathogenesis are still not fully understood (Wang et al., 2024).

Bioinformatics approaches are crucial for analyzing complex biological networks and understanding disease mechanisms. By using genomic, transcriptomic, and proteomic data, bioinformatics allows for the systematic analysis of gene expression patterns and their functional relationships in disease contexts. This study applies bioinformatics methods to examine glycolysis-related genes in IPF. By integrating multi-omics data, we aim to identify key genes and pathways involved in glycolysis that may contribute to the pathogenesis of IPF. These findings could identify potential biomarkers or therapeutic targets for the disease, suggesting that targeting glycolysis-related pathways may offer novel pharmacological strategies for managing IPF.

## Materials and methods

### Data source of bulk RNA sequencing (RNA-seq) and single-cell RNA sequencing (scRNA-seq)

In this study, we used both bulk RNA sequencing (RNA-seq) and single-cell RNA sequencing (scRNA-seq) data to explore gene expression profiles related to IPF. The bulk RNA-seq data were obtained from the Gene Expression Omnibus (GEO) (https://www.ncbi.nlm.nih.gov/gds/) database (Goddard et al., 2000; Jorge et al., 1978). The GSE70866 dataset contains 196 samples, including 176 IPF samples and 20 normal samples. Gene expression profiling was carried out on cells from bronchoalveolar lavage (BAL) fluid collected during regular clinical procedures, with normal samples from healthy individuals undergoing bronchoscopy for reasons not related to IPF to ensure proper controls. The GSE218997 dataset consists of 137 samples from the lung tissues of male C57 mice. The subjects were divided into two age groups: young mice aged 8–12 weeks and aged mice at 21 months. Among these samples, 59 were assigned to the bleomycin (BLM)-induced fibrosis group, and the remaining 78 were used as control samples. Single-cell RNA-seq data on IPF and normal samples were also acquired from the GEO database (GSE128033). Lung tissues were collected during transplant surgeries in line with approved protocols, enabling a detailed examination of gene expression at the single-cell level (Morse et al., 2019). However, detailed patient characteristics like age, sex, and treatment status were not provided in these datasets and thus were not considered in our analysis. Specifically, the datasets did not indicate whether IPF patients were undergoing any treatments at the time of sample collection, so the potential effects of treatment on the studied pathways could not be evaluated. Our main focus was to identify gene expression patterns associated with IPF itself (Supplementary Table 1). GRGs were obtained from the Molecular Signatures Database v7.0 (MSigDB) (https://software.broadinstitute.org/gsea/msigdb/index.jsp) (Supplementary Table 2; Supplementary Data 1) (Male et al., 1987; Zacho et al., 1975; Wheeler, 1973).

### Function enrichment analysis of GRGs

The “limma” R package was used to analyze the expression differences of GRGs in IPF (Burch and Varkey, 1985). Differentially expressed genes were identified using thresholds of log fold-change (logFC) >1 and adjusted p-value (adj.P.Val) <0.05 to ensure robust selection of significant genes. Gene Ontology (GO) and Kyoto Encyclopedia of Genes and Genomes (KEGG) (https://www.kegg.jp/) pathway enrichment analyses were conducted using the “clusterProfiler” R package (Fenster and Downey, 2000; Vobecky et al., 1988). The threshold for each analysis was set at *P* < 0.05.

### IPF characteristic genes screening

The least absolute shrinkage and selection operator (LASSO) regression was performed under 10-fold cross-validation for deeper disease-specific gene selection. The optimal λ generated a minimum cross-validation error (Friedman et al., 2010). We also applied the support vector machine-recursive feature elimination (SVM-RFE) model using the “SVM” R package. In this model, each feature’s score was sorted, and the next iteration was performed, followed by removing the minimum value until the best features were selected. Glycolysis-related differentially expressed genes (GRDEGs) for IPF were identified by overlapping the results of the above algorithms. Moreover, the diagnostic value of GRDEGs was assessed by calculating receiver operating characteristic (ROC) curves using the “pROC” R package (Robin et al., 2011).

### Gene regulatory network and potential therapeutic drug prediction

Gene regulatory network analysis comprises two main components: miRNAs and transcription factors (TFs). Then, the intersection between differential genes was identified in the DEG-miRNA and TF-DEG regulatory networks using NetworkAnalyst (https://www.networkanalyst.ca/home.xhtml) (Xia et al., 2015). TarBase (https://www.microrna.gr/tarbase) and miRTarBase (https://mirtarbase.cuhk.edu.cn/) were used to explore DEG-miRNA interaction networks (Karagkouni et al., 2018). The JASPAR database (https://software.broadinstitute.org/gsea/msigdb/index.jsp) was used to analyze TF-DEG interaction networks (Fornes et al., 2020; Vergoulis et al., 2012; Huang et al., 2020). The DGIdb database (https://www.dgidb.org/) was used to identify potential therapeutic agents for IPF (Miyadera and Iwai, 1964).

### Prognostic model construction

Candidate GRDEGs were used in the univariate Cox and LASSO regressions to construct the prognostic signature using the “glmnet” R package. The formula risk score = (each gene’s expression × corresponding coefficient) was used to classify IPF patients. Patients with a risk score above the median were placed in the high-risk group, and the rest were placed in the low-risk group. The “timeROC” R package was used to construct 1-, 2- and 3-year ROC curves analyses, and the results were quantified by the area under the ROC curve (AUC). Meanwhile, the Kaplan–Meier survival analysis was conducted using ‘‘survival” and ‘‘survminer” R packages. The universal datasets were divided into train and test sets, keeping the ratio to about 1:1. The above analyses were conducted for the whole, train, and test sets.

### Single gene set enrichment analysis and gene set variation analysis

The GSEA was conducted using the “GSEABase” R package (Subramanian et al., 2005). According to the log_2_[Fold Change (FC)] value from the differential analysis, genes were ranked from high to low to be defined as the test gene set. Then, the KEGG signaling pathway set was used to detect the possible relevance between hub genes and the gene set.

This analysis was complemented using the “GSVA” R package (Hänzelmann et al., 2013). The KEGG pathway set was used to conduct the GSVA of hub genes. The “limma” R package was used to identify the GSVA score differences between high and low-expression samples, with *P* < 0.05 as the cut-off. The pathway was classified as active if t > 0 in the high expression group and if t < 0 in the low expression group.

### Immune infiltration analysis

CIBERSORT (Cell-type Identification By Estimating Relative Subsets Of RNA Transcripts) is a computational method for estimating the composition of complex tissues from gene expression profiles (Newman et al., 2019). In this study, we applied CIBERSORT to analyze the proportion of 22 types of infiltrating immune cells in the GSE70866 dataset, which comprises gene expression profiles derived from BAL fluid samples of both IPF patients and healthy controls. Additionally, Pearson correlation coefficients were calculated to quantify the association between the relative abundance of immune cells and the expression levels of hub genes.

### Processing and analysis of single cell transcriptome data

We obtained the raw data from the GEO database (GSE128033) and processed it using the “Seurat” R package (Hao et al., 2021). The data preprocessing steps included normalization, scaling, and cell clustering, resulting in the identification of four predominant cell types. To ensure data quality, we applied specific criteria: nFeature_RNA >200, nFeature_RNA <5500, percent.mt <10, nCount_RNA >1000, nCount_RNA <35,000 (Ilicic et al., 2016). Single cells were extracted while excluding doublets and dead cells based on these criteria. Next, we performed principal component analysis (PCA) on the highly variable genes and reduced the dimensions of the data. The resulting clusters were identified using the functions “FindNeighbors,” “FindClusters,” and ‘runTSNE'.

### Validation of expression of prognostic genes in mouse model

We initially identified 14 glycolysis-related genes using LASSO and SVM-RFE. LASSO regression then narrowed the list to seven genes with prognostic significance, minimal redundancy, and reduced multicollinearity. To validate these genes *in vivo*, we used a bleomycin (BLM)-induced pulmonary fibrosis model in 24 male C57 mice (8–12 weeks old), randomly assigned to either the PBS group (n = 12) or the BLM group (n = 12; 2.5 U/kg, intratracheal). After 21 days, pulmonary fibrosis was confirmed by histology (HE and Masson staining) and Western blot (Col1a1, Fibronectin). We subsequently measured the expression of the seven selected genes by qPCR (primer sequences in Supplementary Table 3). All animal procedures were approved by the Ethics Committee of Wuhan University (No. WP20220442).

### Statistical analysis

R software (version 4.0.3) was used for statistical analyses and visualization of results. ALL group’s difference calculated by Mann-Whitney U test. A *P* < 0.05 was considered statistically significant. Significance correlation coefficients were defined by absolute values >0.2 and *P* < 0.05.

## Results

### Identification and functional analyses of GRDEGs

**FIGURE 1:**
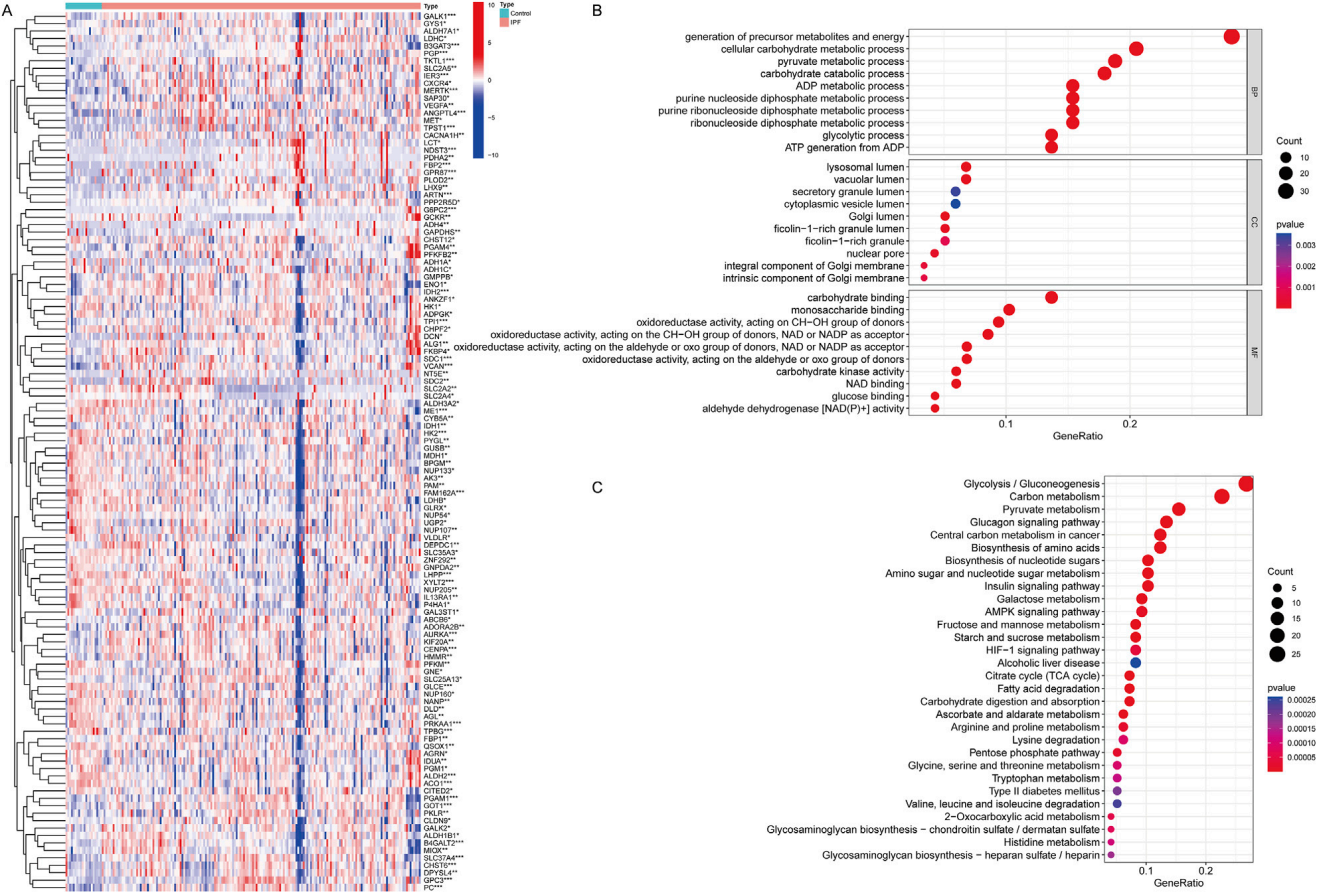
Differentially expressed genes of Glycolysis in IPF and functional analysis for these genes. **(A)** Heatmap showing the differential expression of GRGs between IPF patients and healthy controls. Each row represents a gene, and each column represents a sample. Red indicates upregulation, and blue indicates downregulation. Distinct clustering of gene expression profiles highlights significant differences between the two groups. *P < 0.05, **P < 0.01, ***P < 0.001. Mann-Whitney U test. **(B)** Gene Ontology (GO) enrichment analysis of differentially expressed GRGs, categorized into biological processes (BP), cellular components (CC), and molecular functions (MF). **(C)** KEGG pathway enrichment analysis of differentially expressed GRDEGs. (Con:Healthy Volunteer, IPF: Idiopathic Pulmonary Fibrosis patients).

The analysis of GRGs in the GSE70866 dataset revealed significant expression differences between IPF patients and healthy controls, including 63 upregulated genes and 55 downregulated genes. Key glycolytic enzymes, such as ***ENO1***, were significantly upregulated in IPF (*P* = 0.028), indicating enhanced glycolytic activity, while genes involved in oxidative metabolism, such as ***DLD*** and ***ADH1C***, were markedly downregulated (*P* < 0.05), suggesting impaired energy homeostasis (Figure 1A; Supplementary Data 2). GO analysis further highlighted their localization to key cellular compartments, including the lysosomal lumen and vascular lumen, emphasizing roles in cellular transport and energy regulation (Figure 1B). Functional enrichment analysis demonstrated that differentially expressed GRGs were involved in critical processes, including energy generation, carbohydrate metabolism, and oxidative stress regulation, with significant enrichment in biological pathways such as **glycolysis/gluconeogenesis**, ***HIF-1* signaling**, and **carbon metabolism**. These findings indicate a hypoxia-driven metabolic reprogramming in IPF (Figure 1C). Collectively, these results suggest that metabolic alterations in GRGs contribute to fibroblast activation, extracellular matrix remodeling, and disease progression, offering potential targets for therapeutic intervention in IPF.

### GRDEGs were diagnostic genes for IPF

**FIGURE 2:**
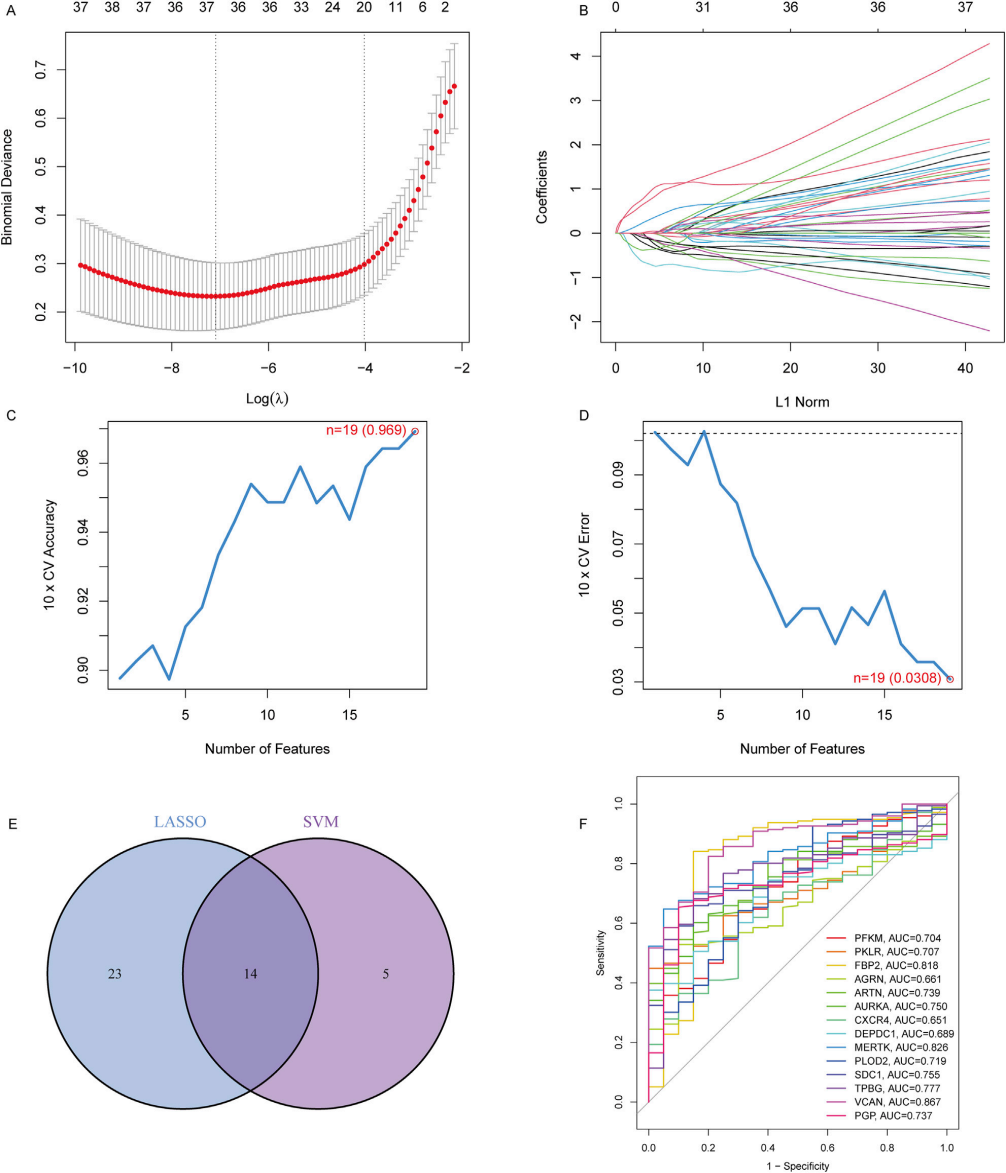
Identification of key diagnostic genes in IPF using LASSO and SVM-RFE regression. **(A)** Binomial deviance curve for LASSO regression. The optimal lambda (λ) value was selected based on the minimum binomial deviance with 10-fold cross-validation, reducing the number of candidate features to 37. **(B)** LASSO coefficient profiles for the 37 features, showing the shrinking of coefficients as the penalty increases. **(C)** Cross-validation accuracy of SVM regression, with the highest accuracy of 0.969 achieved when selecting 19 features. **(D)** Cross-validation error of SVM regression, with the lowest error (0.0308) corresponding to 19 features. **(E)** Venn diagram showing the overlap of selected genes from the LASSO and SVM-RFE models, identifying 14 overlapping genes. **(F)** ROC curves showing the diagnostic performance (AUC) of the 14 signature genes in distinguishing IPF patients from controls.

To identify robust biomarkers for IPF, a combination of LASSO regression and SVM-RFE algorithms was applied to optimize feature selection. LASSO regression analysis, guided by 10-fold cross-validation, selected 37 candidate genes with minimal binomial deviance, as shown in Figure 2A. The corresponding coefficient profiles (Figure 2B) highlight the effect of regularization, where increased λ progressively reduced the number of retained variables. SVM-RFE further refined the selection to 19 genes, achieving the highest cross-validation accuracy (0.969) and the lowest error rate (0.0308), as depicted in Figures 2C, D. The overlap between the LASSO and SVM-RFE methods was visualized in a Venn diagram (Figure 2E), revealing 14 genes shared by both models. These shared genes, including *PFKM, AURKA, CXCR4*, and *SDC1*, were prioritized for their diagnostic potential. ROC curve analysis (Figure 2F) demonstrated the diagnostic performance of each gene, with AUC values ranging from 0.704 (*PFKM*) to 0.887 (*VCAN*), underscoring their utility in distinguishing IPF patients from controls. Overall, this integrated computational approach identified a robust panel of 14 genes with high diagnostic accuracy, providing a basis for further experimental validation and mechanistic exploration in IPF pathogenesis.

### Identification of candidate drugs

**FIGURE 3:**
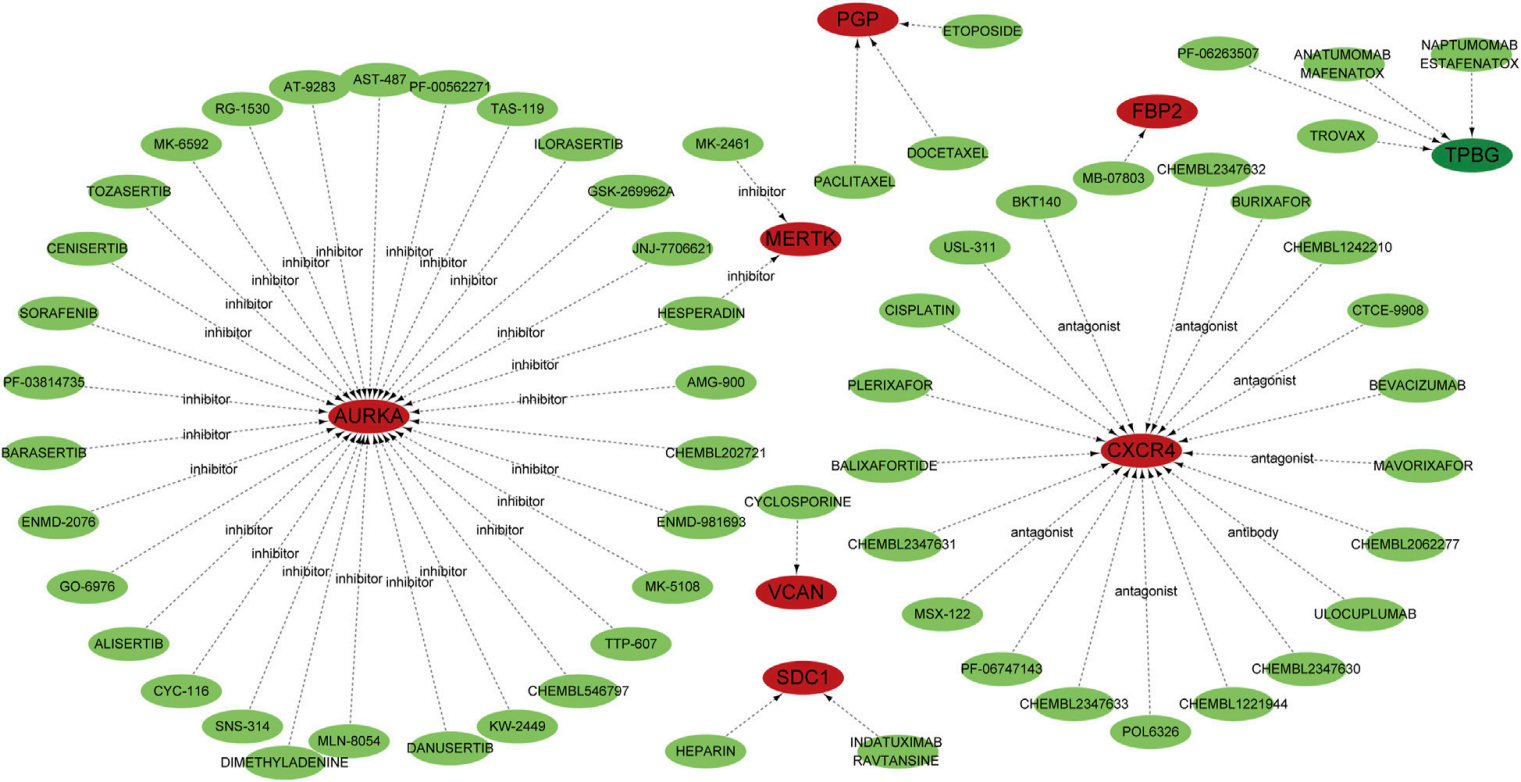
Potential target drugs were predicted using the DGIdb database and are represented by green nodes. Genes are depicted as red nodes, while the dotted lines indicate predicted interactions. Specific drug classes, such as inhibitors, antagonists, and antibodies, are annotated to show the type of interaction with their corresponding genes.

To identify potential therapeutic agents targeting the 14 key genes (*PFKM, PKLR, FBP2, AGRN, ARTN, AURKA, CXCR4, DEPDC1, MERTK, PLOD2, SDC1, TPBG, VCAN*, and *PGP*) implicated in IPF, we used the DGIdb. Through Cytoscape for visualization, the analysis showed a comprehensive network of drug-gene interactions, providing practical insights for targeted therapeutic strategies (Figure 3). *AURKA* was related to a variety of inhibitors, including Tozasertib, Alisertib, and Barasertib, which are known to control mitosis and cellular proliferation (Dickson et al., 2016; Wang-Bishop et al., 2019). *CXCR4*, a crucial chemokine receptor, was connected to antagonists like Plerixafor, Mavorixafor, and CTCE-9908, which might suppress fibrosis by adjusting immune cell migration and inflammation (Hassan et al., 2011; Kwong et al., 2009). *MERTK* was recognized as a target of inhibitors such as Paclitaxel and Cisplatin, indicating its function in apoptosis regulation and tissue remodeling (Choi et al., 2020; Kang et al., 2015). Other notable interactions included *VCAN*, targeted by agents such as Cyclosporine and Balixafortide, which could influence extracellular matrix dynamics (Luo et al., 2023; Choocheep et al., 2010), and *SDC1*, which was linked to drugs like Heparin and Indatuximab, emphasizing its involvement in cell adhesion and matrix interactions (Schönfeld et al., 2018; Floer et al., 2010). *TPBG* was targeted by antibody-based therapies such as Naptumomab, underlining its potential in immune modulation (Sun et al., 2024; Hu et al., 2015). Additionally, metabolic regulators *FBP2* and *PGP* were associated with chemotherapeutic agents such as Etoposide and Docetaxel, suggesting their roles in stress response pathways (Kemper et al., 2004; Roy et al., 2014). These discoveries emphasize the therapeutic significance of the identified genes and their druggable targets, providing a basis for precision medicine approaches in IPF.

### TF-gene and miRNA-gene networks

**FIGURE 4:**
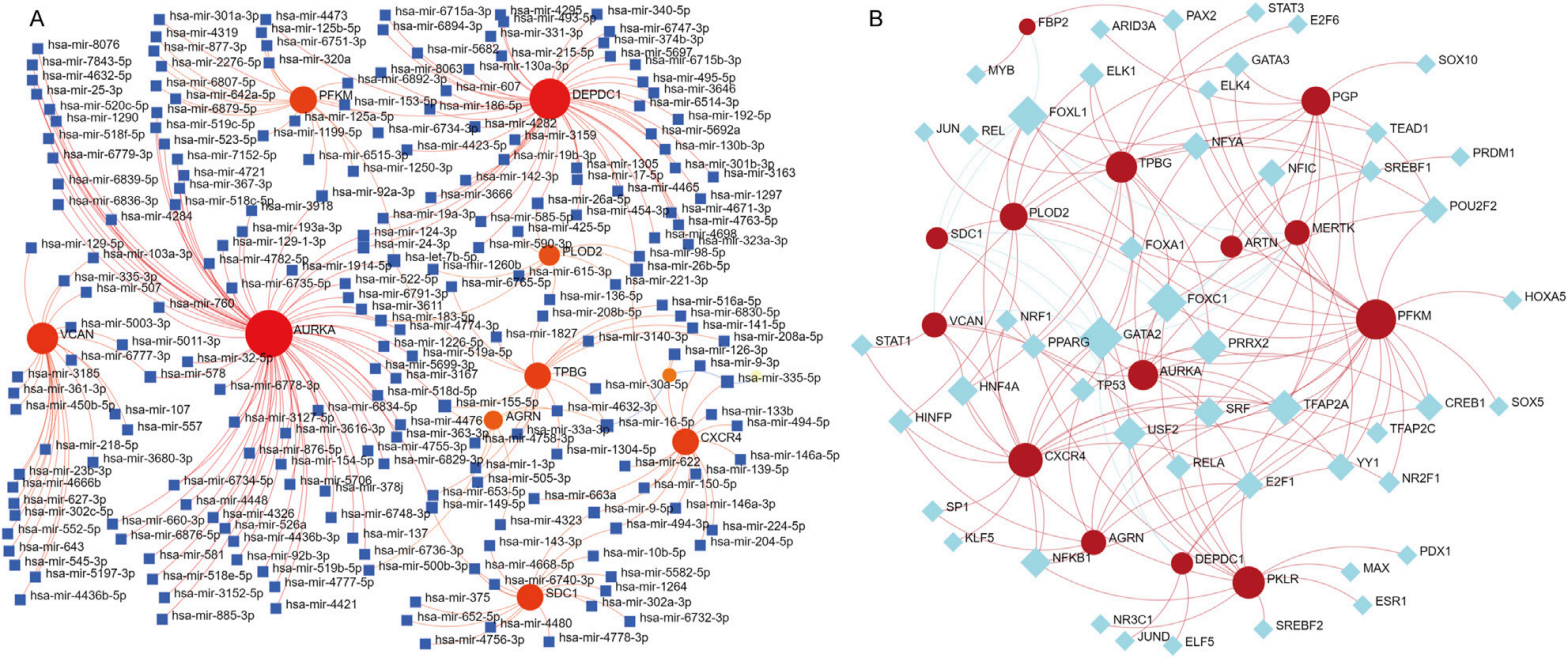
Prediction of miRNA and transcription factor (TF)-RNA interactions for the 14 identified genes. **(A)** miRNA-gene interaction network generated using NetworkAnalyst. Red nodes represent the 14 key genes, blue square nodes represent miRNAs, and red edges indicate predicted regulatory relationships. The network illustrates the potential post-transcriptional regulatory roles of miRNAs in modulating the expression of these genes. **(B)** TF-gene interaction network, where red nodes indicate the key genes and blue diamond nodes represent transcription factors. The red edges represent predicted regulatory interactions, providing insights into the transcriptional regulation of these genes.

To elucidate the regulatory mechanisms of the 14 key genes involved in IPF, we used NetworkAnalyst to construct miRNA-gene and TF-gene interaction networks. These analyses show the complex layers of transcriptional and post-transcriptional regulation that might contribute to the pathogenesis of IPF. The miRNA network found many regulatory miRNAs interacting with the target genes, with *VCAN, AURKA,* and *TPBG* emerging as central hubs. Key miRNAs (Figure Figure4A), such as hsa-mir-125a-5p, hsa-mir-146a-5p, and hsa-mir-335-5p, were predicted to target multiple genes, indicating their roles as important post-transcriptional regulators (Cheng et al., 2024; Zhou et al., 2024; Li et al., 2014). These miRNAs probably affect processes crucial to IPF, including extracellular matrix remodeling, immune modulation, and cellular proliferation. The TF network revealed major transcriptional regulators such as *TP53, STAT1*, and *GATA2*, which are connected to multiple target genes like *CXCR4, VCAN,* and *AURKA* (Figure 4B) (Dauch et al., 2016; Feng et al., 2020; Rugge et al., 2010). Additional TFs like PPARG, RELA, and NRF1 are linked to immune responses, oxidative stress pathways, and metabolism, emphasizing their role in coordinating the gene dysregulation in IPF (Koudstaal and Wijsenbeek, 2023; Tian et al., 2023). Genes like CXCR4 and AURKA are identified as key targets for these regulatory TFs, highlighting their functional significance in IPF pathophysiology (Smadja et al., 2014; Jaffar et al., 2020). These findings offer a detailed regulatory framework for the key genes related to IPF, emphasizing the interaction between miRNAs and TFs in regulating their expression. The networks identify potential upstream regulators, providing valuable insights into disease mechanisms and potential therapeutic targets.

### Establishment of the GRDEG prognostic signature

**FIGURE 5:**
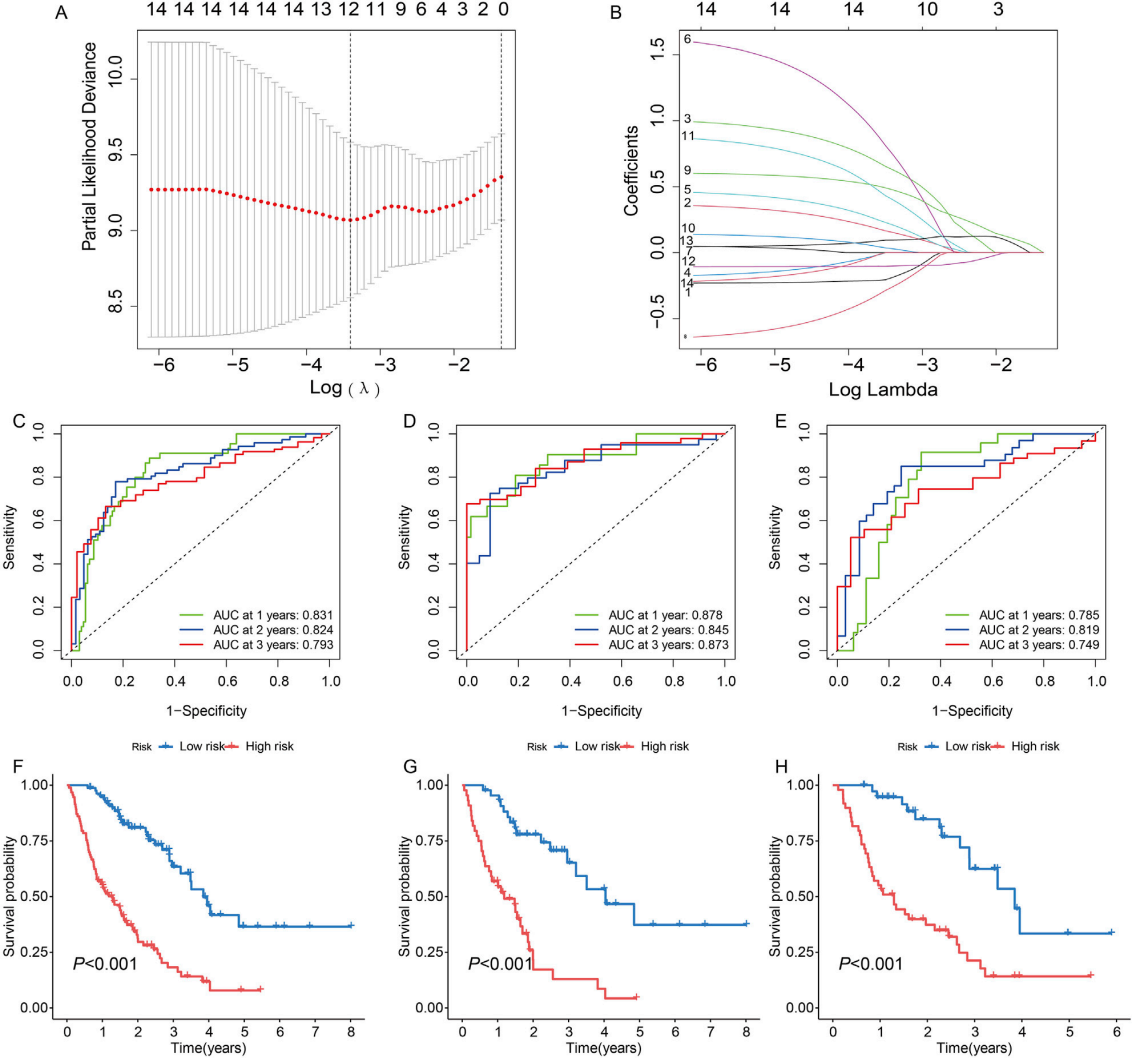
Construction and validation of a survival model based on 14 genes in idiopathic pulmonary fibrosis (IPF) patients using LASSO regression. **(A)** Partial likelihood deviance for LASSO regression. The optimal lambda (λ) value was selected based on the minimum deviance. **(B)** Coefficient profiles of the genes as a function of the log-transformed lambda (λ) value. **(C, F)** Model performance for the entire cohort: **(C)** Receiver operating characteristic (ROC) curves with AUC values of 0.831, 0.824, and 0.793 for predicting 1-, 2-, and 3-year survival, respectively. **(F)** Kaplan-Meier survival curves showing significant differences in survival between high- and low-risk groups (*P* < 0.001). **(D, G)** Performance in the training set: **(D)** ROC curves with AUC values of 0.878, 0.845, and 0.873 at 1, 2, and 3 years, respectively. **(G)** Kaplan-Meier curves indicating significant survival differences between risk groups (*P* < 0.001). **(E, H)** Validation in the testing set: **(E)** ROC curves demonstrating AUC values of 0.785, 0.819, and 0.749 at 1, 2, and 3 years, respectively. **(H)** Kaplan-Meier analysis confirming significant survival differences between high- and low-risk groups (*P* < 0.001).

**FIGURE 6:**
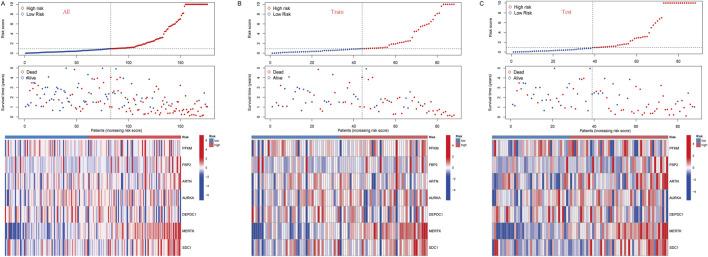
**(A)** In all groups, risk score and survival status of low-and high-risk patients, and heatmap of 7 glycolysis-related genes expression levels. **(B)** In the train group, risk score and survival status of low-and high-risk patients, and heatmap of 7 glycolysis-related genes expression levels. **(C)** In the test group, risk score and survival status of low-and high-risk patients, and heatmap of seven glycolysis-related genes expression levels.

We constructed a survival model using LASSO regression based on 7 genes (*PFKM, FBP2, ARTN, AURKA, DEPDC1, MERTK, and SDC1*) selected from an initial pool of 14, integrating survival data from IPF patients (Figures 5A, B). The results demonstrate the model’s predictive accuracy and effective risk stratification. In the overall cohort, the model demonstrated predictive performance, with AUC values of 0.831, 0.824, and 0.793 at 1, 2, and 3 years, respectively (Figure 5C). Kaplan-Meier survival analysis revealed significant survival differences between high- and low-risk groups (*P* < 0.001, Figure 5F). After a 1:1 split into training and testing cohorts, the model performed well in the training set, achieving AUC values of 0.878, 0.845, and 0.873 at 1, 2, and 3 years, respectively (Figure 5D). Survival analysis confirmed the model’s ability to stratify patients effectively (*P* < 0.001, Figure 5G). Validation in the testing cohort yielded consistent AUC values of 0.785, 0.819, and 0.749 at 1, 2, and 3 years, respectively (Figure 5E). Kaplan-Meier analysis further demonstrated significant survival differences between risk groups (*P* < 0.001, Figure 5H). Risk scores, derived from the survival model, stratified patients into high- and low-risk groups. Risk scores correlated positively with mortality, as visualized in the scatterplots and gene expression heatmaps. In the overall cohort (Figure 6A), as well as the training (Figure 6B) and testing subsets (Figure 6C), higher risk scores were associated with a greater number of deaths. The heatmaps further demonstrated the differential expression of the 7 genes across risk groups, reinforcing their contribution to the model.

### Marker genes were connected with various IPF-related pathways

**FIGURE 7:**
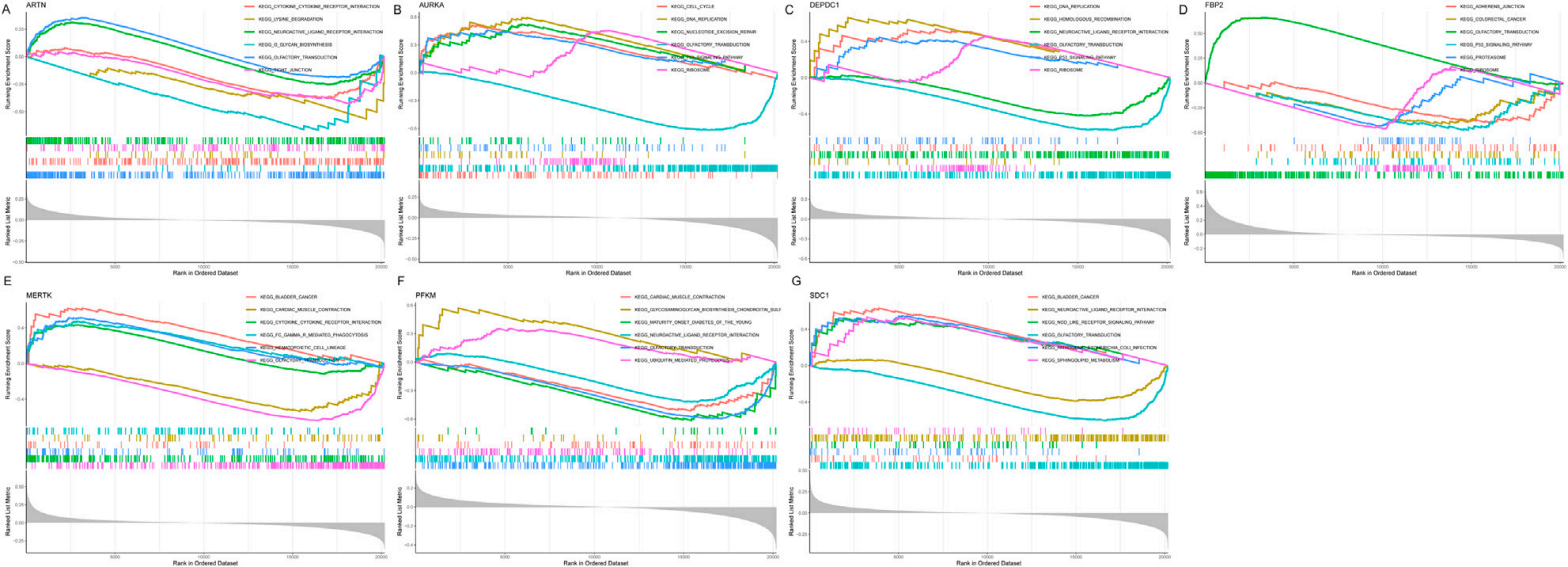
Figure: Gene Set Enrichment Analysis (GSEA) of the seven selected genes in idiopathic pulmonary fibrosis (IPF). **(A–G)** GSEA results for the seven genes: *ARTN* **(A)**, *AURKA* **(B)**, *DEPDC1* **(C)**, *FBP2* **(D)**, *MERTK* **(E)**, *PFKM* **(F)**, and *SDC1* **(G)**. Each panel displays the enrichment plots for significantly associated KEGG pathways, ranked by enrichment score.

To further explore the potential function of marker genes to distinguish IPF from normal samples, we conducted a single-GSEA-KEGG pathway analysis. The top six pathways enriched for each marker gene were presented in Figures 7A–G. *ARTN* was enriched in cytokine-cytokine receptor interaction, lysine degradation, neuroactive ligand-receptor, glycan biosynthesis, olfactory transduction, and tight junction. *AURKA* was enriched in the cell cycle, DNA replication, nucleotide excision repair, olfactory transduction, *P53* signaling pathway, and the ribosome. *FBP2* was also related to the *P53* signaling pathway, olfactory transduction, and ribosome. Besides, *FBP2* was enriched in other pathways, including adherens junction, colorectal cancer, and the proteasome. *DEPDC1* was enriched in DNA replication, homologous recombination, neuroactive ligand-receptor interaction, olfactory transduction, *P53* signaling pathway, and the ribosome. *MERTK* was enriched in bladder cancer, cardiac muscle contraction, cytokine-cytokine receptor interaction, Fc gamma R-mediated phagocytosis, hematopoietic cell lineage, and olfactory transduction. *PFKM* was enriched for cardiac muscle contraction, glycosaminoglycan biosynthesis, chondroitin sulfa-, maturity-onset diabetes of the young, neuroactive ligand-receptor interaction, olfactory transduction, and ubiquitin-mediated proteolysis. Finally, *SDC1* was enriched in bladder cancer, neuroactive ligand-receptor interaction, nod-like receptor signaling pathway, olfactory transduction, pathogenic *Escherichia coli* infection, and sphingolipid metabolism.

**FIGURE 8:**
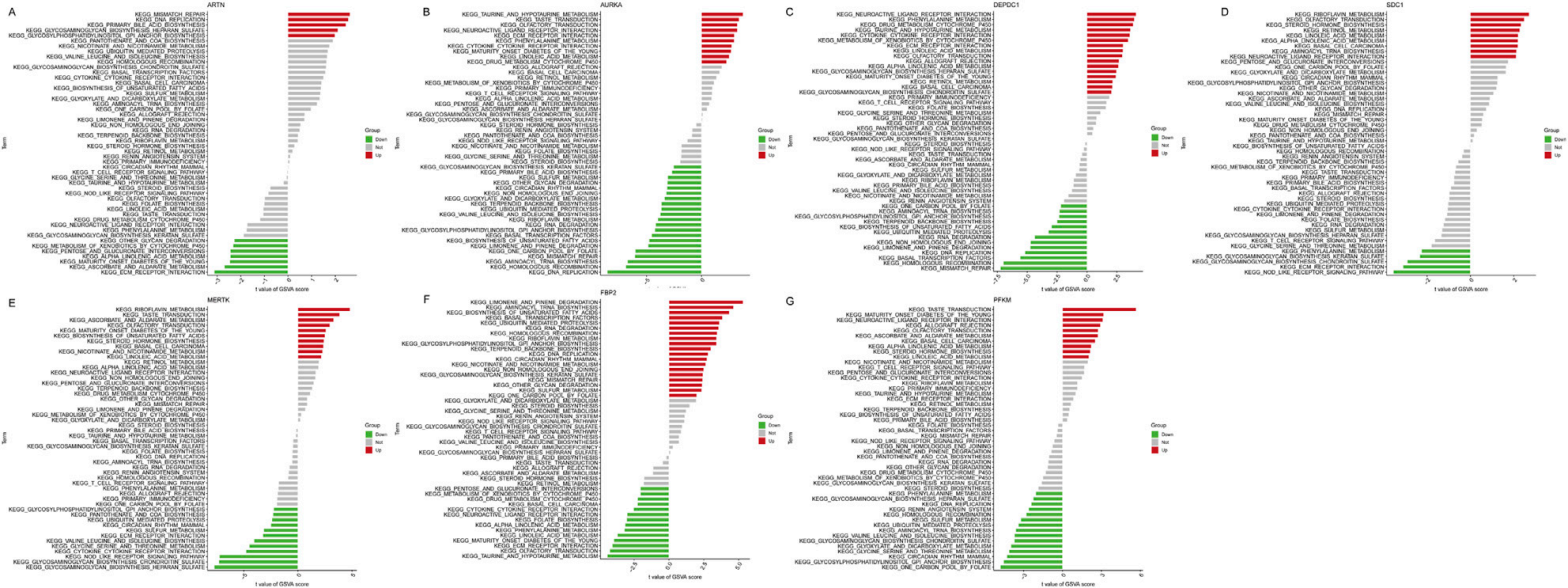
Figure: Gene Set Variation Analysis (GSVA) of seven key genes in idiopathic pulmonary fibrosis (IPF). **(A–G)** GSVA results for *ARTN* **(A)**, *AURKA* **(B)**, *DEPDC1* **(C)**, *FBP2* **(D)**, *MERTK* **(E)**, *PFKM* **(F)**, and *SDC1* **(G)**. Each plot displays the KEGG pathways significantly associated with each gene, categorized as upregulated (red), downregulated (green), or not significantly changed (gray). The x-axis shows the t-values of the GSVA scores for each pathway.

Moreover, the GSVA showed that the low *ARTN* expression might induce IPF by activating ECM receptor interaction, ascorbate and aldarate metabolism, and maturity-onset diabetes of the young, while *ARTN* overexpression activated mismatch repair, DNA replication, and primary bile acid biosynthesis (Figure 8A). Moreover, *AURK* upregulation activated the taurine and hypotaurine metabolism and olfactory transduction, while Its downregulation activated DNA replication and homologous recombination (Figure 8B). High *DEPDC1* expression activated neuroactive ligand-receptor interaction and phenylalanine metabolism, while low *DEPDC1* expression activated DNA replication (Figure 8C). Riboflavin metabolism, steroid hormone biosynthesis, and linoleic acid metabolism were enriched in the high *SDC1* expression group, while NOD-like receptor signaling, ECM receptor interaction, glycosaminoglycan biosynthesis chondroitin sulfate, and glycosaminoglycan biosynthesis keratan sulfate were enriched in the low-expression group (Figure 8D). High *MERTK* expression was related to riboflavin metabolism, while its downregulation was associated with glycosaminoglycan biosynthesis heparan sulfate- (Figure 8E). Notably, for *FBP2,* various pathways related to IPF pathogenesis were enriched, including limonene and pinene degradation, aminoacyl tRNA biosynthesis, and biosynthesis of unsaturated fatty acids. Low *FBP2* expression was related to taurine and hypotaurine metabolism and olfactory transduction (Figure 8F). Furthermore, *PFKM*, whose expression was limited in IPF, was more closely related to one carbon pool by folate, glycosylphosphatidylinositol GPI anchor biosynthesis, and the mammal circadian rhythm. Noteworthy, high *PFKM* expression activated the pathways such as taste transduction, maturity-onset diabetes of the young, neuroactive ligand-receptor interaction, and allograft rejection that might induce IPF (Figure 8G).

### Immune landscape analysis

**FIGURE 9:**
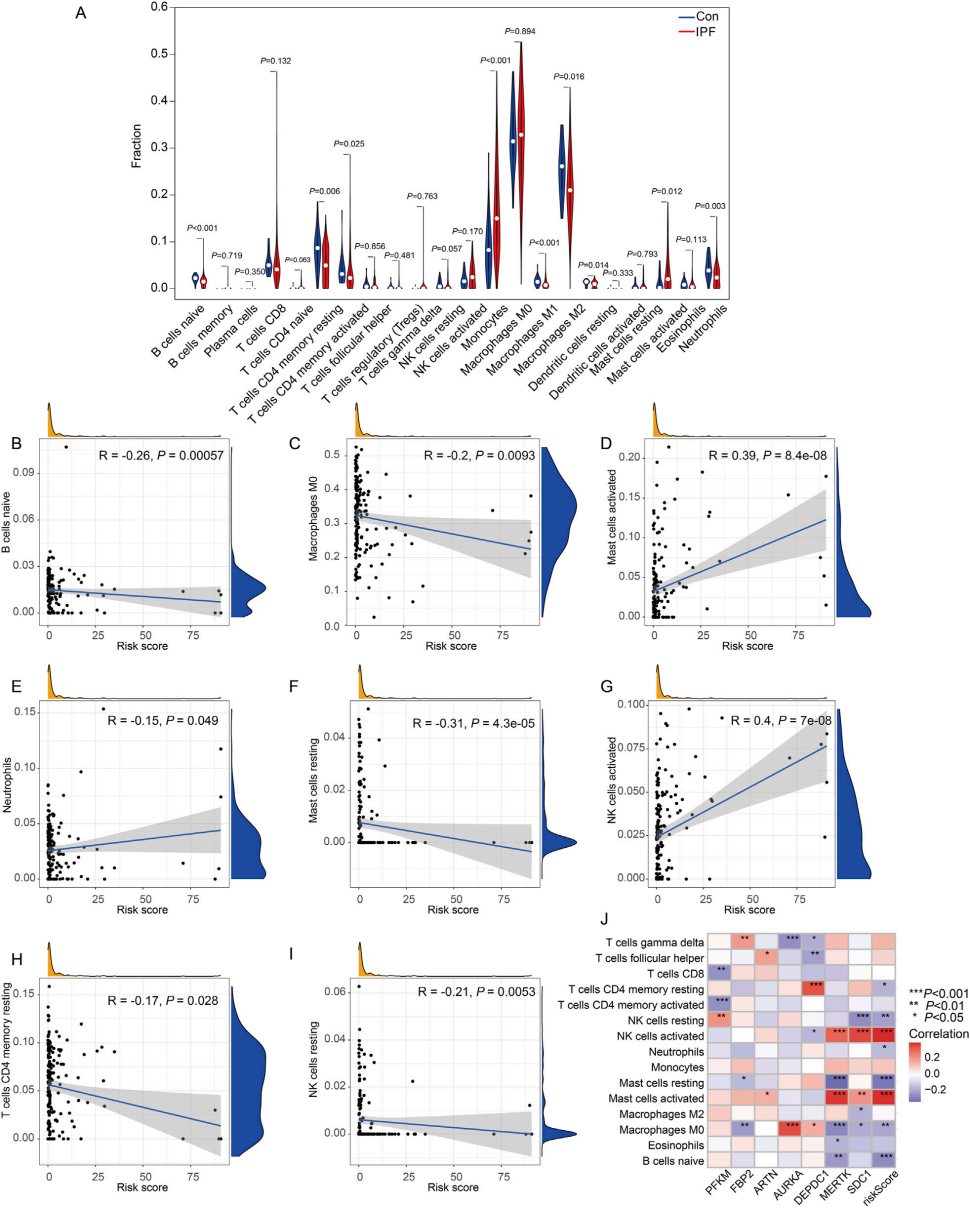
Immune cell infiltration analysis and correlation with the risk score in IPF. **(A)** Violin plots representing the proportion of immune cell subtypes in the control group (blue) and treatment group (red). **(B–I)** Scatter plots showing the correlation between immune cell proportions and the risk score: **(B)** B cells naïve demonstrated a negative correlation (R = −0.26, *P* = 0.00057). **(C)** Macrophages M0 also showed a significant negative correlation (R = −0.2, *P* = 0.0093). **(D)** Mast cells activated were positively correlated with the risk score (R = 0.39, *P* = 8.4e-08). **(E)** Neutrophils displayed a negative correlation (R = −0.15, *P* = 0.049). **(F)** Mast cells resting exhibited a negative correlation (R = −0.31, *P* = 4.3e-05). **(G)** NK cells activated were positively correlated with the risk score (R = 0.4, *P* = 7e-08). **(H)** T cells CD4 memory resting showed a negative correlation (R = −0.17, *P* = 0.028). **(I)** NK cells resting also demonstrated a negative correlation (R = −0.21, *P* = 0.0053). Each scatter plot includes density distributions for the proportion of immune cells and the risk score along the axes, along with a regression line to visualize trends. **(J)** Heatmap illustrates the correlation between the expression levels of key genes (rows) and immune cell infiltration levels (columns). The correlation coefficients are color-coded, with significant associations denoted by asterisks (*P < 0.05, **P < 0.01, ***P < 0.001, Con:Healthy Volunteer, IPF: Idiopathic Pulmonary Fibrosis patients).

Then, we evaluated the relationship between the infiltration of immune cells in IPF and normal samples (Figure 9A). Nine of the 22 immune cell types differed between the two groups: naive B cells, resting CD4 memory T cells, activated CD4 memory T cells, monocytes, M1 macrophages, M2 macrophages, activated dendritic cells, activated mast cells, and neutrophils. Next, we assessed the relationship between immune cells and risk scores using Pearson correlation analysis (Figures 9B–I). Naive B cells (r = −0.26, *P* = 0.00057), M0 macrophages (r = −0.2, *P* = 0.0093), resting mast cells (r = 0.39, *P* = 8.4e-08), CD4 memory T cells (r = −0.15, *P* = 0.049), and NK memory T cells (r = 0.4, *P* = 7e-08) were negatively correlated with the risk score. Meanwhile, activated mast cells, neutrophils, and activated NK cells were positively associated with risk scores. Pearson’s correlation analysis also revealed the relationships between immune cells and hub genes. *MERTK* had a positive correlation with activated mast cells and a negative correlation with resting mast cells. *SDC1* had a positive correlation with activated NK cells and negative correlations with resting NK cells. *AURKA* was positively correlated with M0 macrophages and negatively correlated with gamma delta T cells. *DEPDC1* was positively correlated with resting CD4 memory T cells. *PFKM* was negatively correlated with activated CD4 memory T cells (Figure 9J).

### Identification of hub gene expression levels and diagnostic value

**FIGURE 10:**
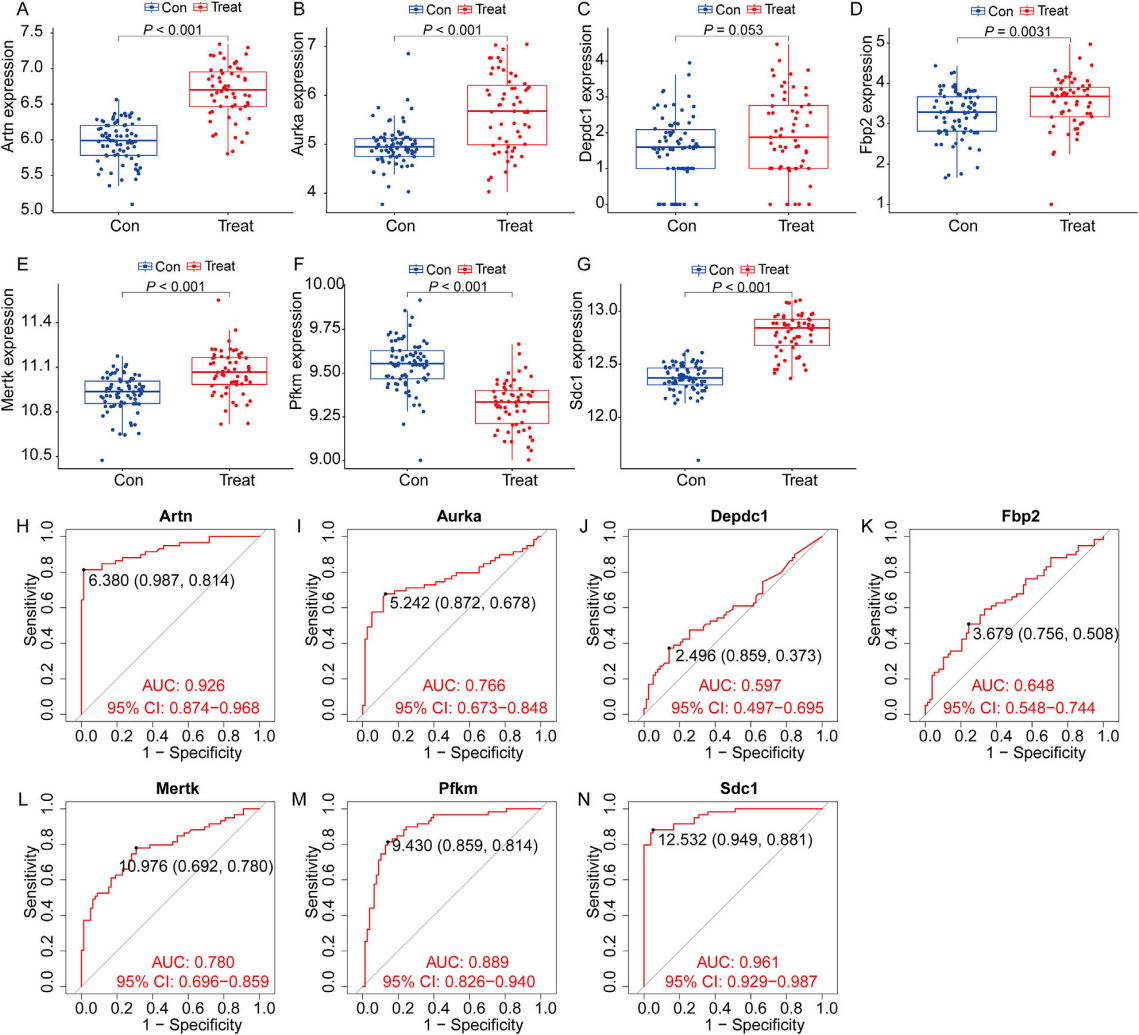
Expression of the target genes and diagnostic value in the verification set. Validation of the expression trends and diagnostic value of seven key genes using the external dataset GSE218997. **(A–G)** Differential expression analysis for the seven genes (*Artn, Aurka, Depdc1, Fbp2, Mertk, Pfkm*, and *Sdc1*) between the control group (Con, blue) and treatment group (Treat, red). Significant differences were observed for most genes (*P* < 0.05), confirming consistent expression trends across groups (All data arepresented as the mean ± IQR. *P < 0.05, **P < 0.01, ***P < 0.001. Mann-Whitney U test). **(H–N)** Receiver Operating Characteristic (ROC) curve analysis evaluating the diagnostic value of the seven genes for identifying IPF. **(H)**, *Artn* **(I)**, *Aurka* **(J)**, *Depdc1* **(K)**, *Fbp2* **(L)**, *Mertk* **(M)**, *Pfkm* and *Sdc1* **(N)**. (Con:Healthy mice, Treat: bleomycin (BLM)-induced pulmonary fibrosis model in C57BL/6 mice).

To validate the expression trends and diagnostic value of the seven selected genes in IPF, we analyzed an external dataset (GSE218997). Differential expression analysis revealed that *Artn, Aurka, Mertk, Pfkm,* and *Sdcl* were significantly upregulated in the lung tissue of mice induced with bleomycin compared to saline controls (*P* < 0.001, Figures 10A, B, E–G), while *Fbp2* also showed increased expression with moderate statistical significance (*P* = 0.0031, Figure 10D). *Depdc1* exhibited a marginally higher expression trend in bleomycin groups, though this did not reach statistical significance (*P* = 0.053, Figure 10C). These results align with the findings from our primary dataset, supporting the robustness of these genes' expression patterns. ROC analysis further confirmed the diagnostic value of these genes. *Artn* (AUC: 0.926, Figure10H), *Sdcl* (AUC: 0.961, Figure10N), and *Pfkm* (AUC: 0.889, Figure10M) demonstrated excellent diagnostic accuracy, while *Aurka* (AUC: 0.766, Figure10I) and *Mertk* (AUC: 0.780, Figure10L) showed good performance. *Fbp2* and *Depdc1* had lower diagnostic utility, with AUCs of 0.648 and 0.597, respectively(Figures 10K, J).These findings further validate the clinical relevance of the survival model and its utility for IPF diagnosis and prognosis.

### Hub gene expression levels in scRNA-seq

**FIGURE 11:**
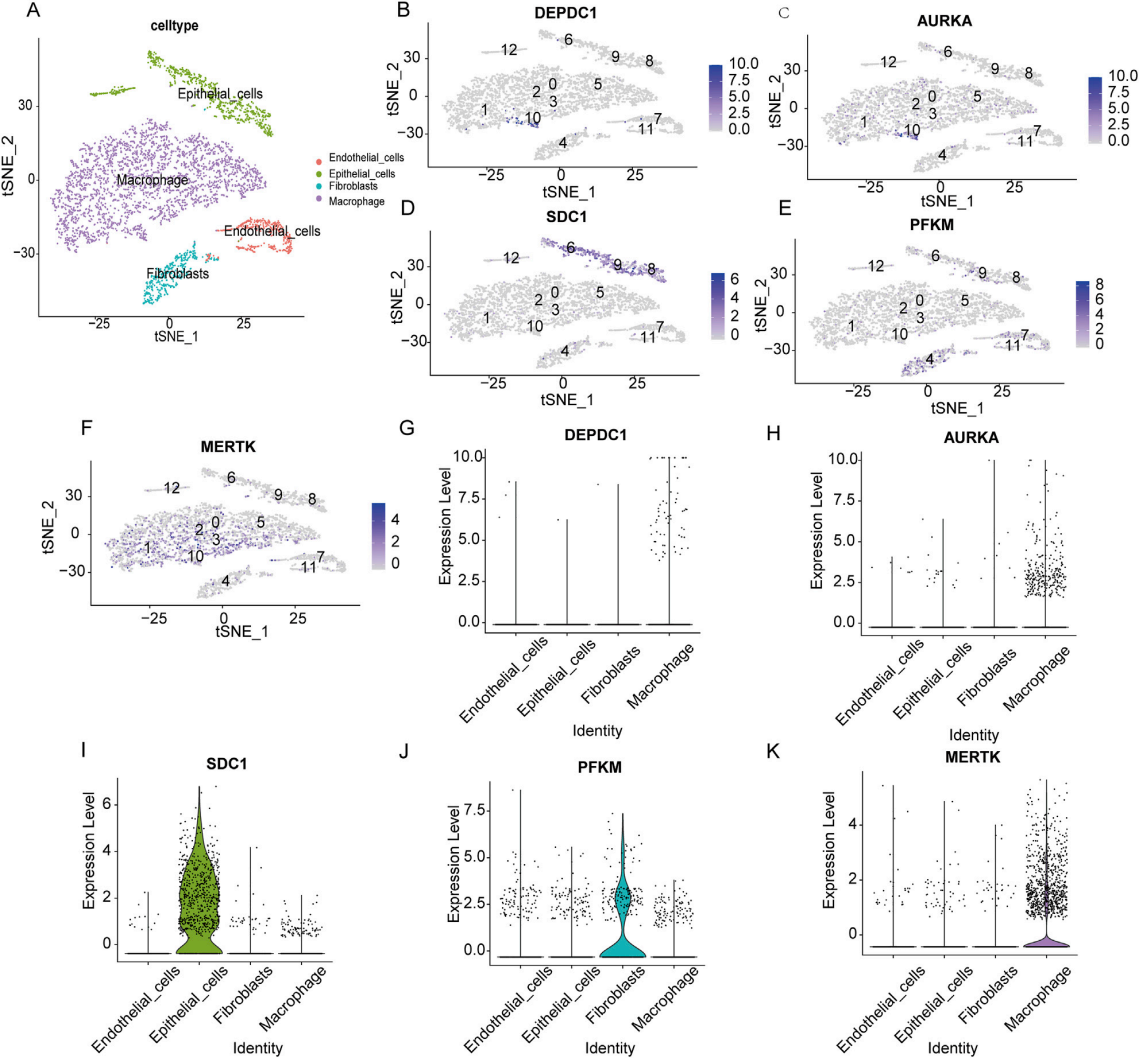
Single-cell RNA sequencing analysis of the expression patterns of key genes across distinct cell types. **(A)** t-SNE plot visualizing the distribution of major cell populations, including endothelial cells (red), epithelial cells (green), fibroblasts (blue), and macrophages (purple). **(B–F)** t-SNE feature plots displaying the expression levels of *DEPDC1* **(B)**, *AURKA* **(C)**, *SDC1* **(D)**, *PFKM* **(E)**, and *MERTK* **(F)** across the identified cell clusters. Higher expression intensities are shown in darker colors. **(G–K)** Violin plots showing the expression levels of *DEPDC1* **(G)**, *AURKA* **(H)**, *SDC1* **(I)**, *PFKM* **(J)**, and *MERTK* **(K)** in endothelial cells, epithelial cells, fibroblasts, and macrophages.

Single-cell RNA sequencing analysis was performed to investigate the cell-type-specific expression patterns of the selected genes in IPF. The t-SNE plot (Figure 11A) identified major cell populations, including epithelial cells, fibroblasts, macrophages, and endothelial cells, providing a comprehensive cellular landscape. Gene expression mapping (Figures 11B–F) revealed distinct cellular localization for each gene. *DEPDC1* (Figure 11B) and *AURKA* (Figure 11C) showed expression in macrophages, suggesting their roles in inflammatory and immune responses. *SDC1* (Figure 11D) was predominantly expressed in epithelial cells, indicating its involvement in maintaining epithelial integrity and extracellular matrix interactions. *PFKM* (Figure 11E) and *MERTK* (Figure 11F) were primarily expressed in fibroblasts and macrophages, implicating them in metabolic pathways and tissue remodeling processes. Violin plots (Figures 11G–K) further quantified gene expression across cell types, confirming these patterns. *DEPDC1* and *AURKA* exhibited significantly higher expression in macrophages, reinforcing their roles in immune regulation. *SDC1* was highly expressed in epithelial cells, while *PFKM* and *MERTK* were enriched in fibroblasts and macrophages, aligning with their respective roles in metabolism and reparative functions. These findings provide a detailed characterization of the cellular distribution of these genes, highlighting their contributions to IPF pathophysiology and their potential as cell-type-specific therapeutic targets.

### Validation of expression of prognostic genes in bleomycin-induced pulmonary fibrosis mouse model

**FIGURE 12:**
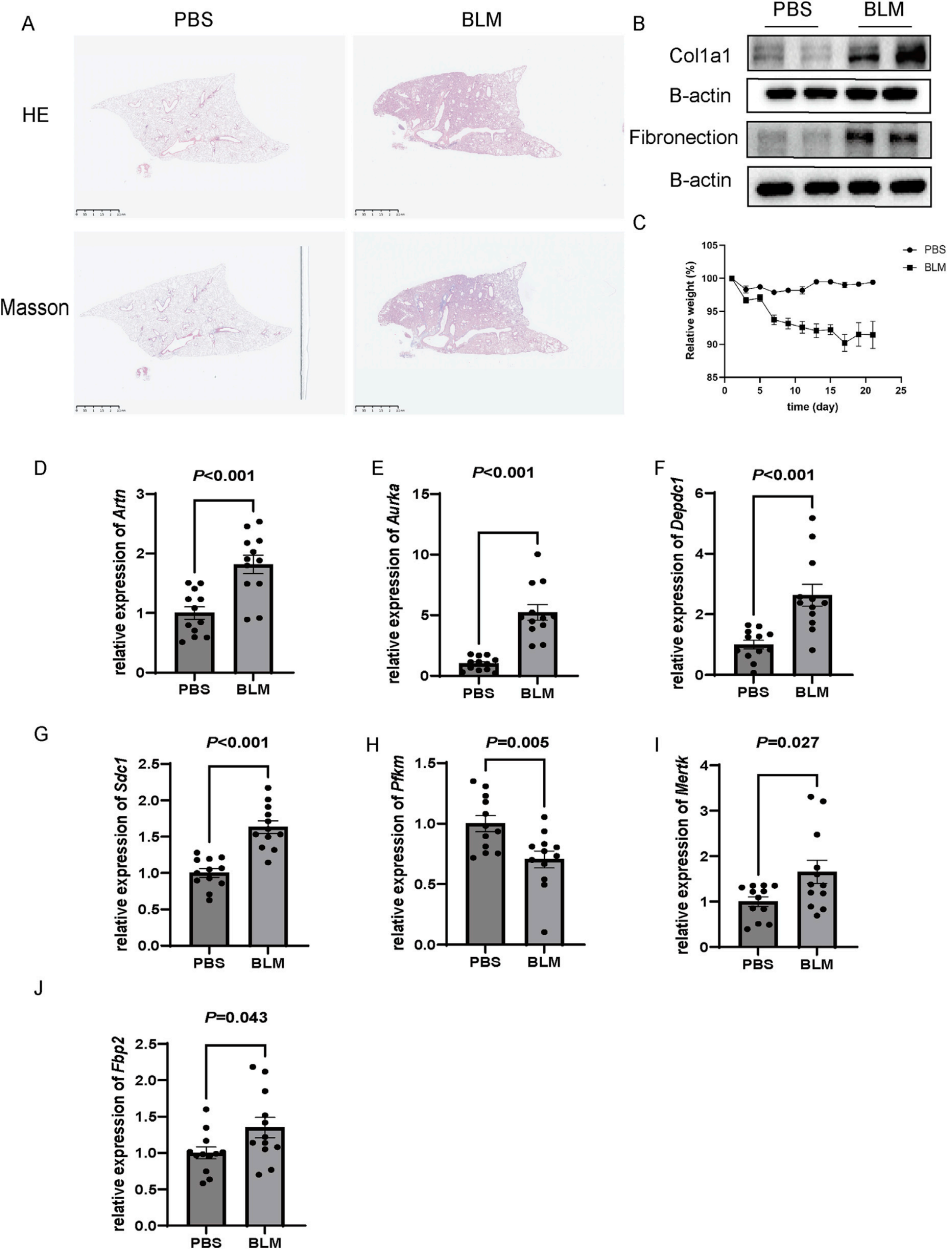
Validation of gene expression in a bleomycin (BLM)-induced pulmonary fibrosis model in C57BL/6 mice. **(A)** Representative histological images of lung tissues stained with Hematoxylin and Eosin (H&E) and Masson’s trichrome. BLM-treated mice exhibited extensive fibrosis and collagen deposition compared to the PBS-treated control group. Scale bar: 2.5 mm. **(B)** Western blot analysis of fibrotic markers Col1a1 and fibronectin in lung tissues, showing increased protein expression in the BLM group. β-actin was used as a loading control. **(C)** Relative body weight changes over 25 days in PBS and BLM-treated mice, with significant weight loss observed in the BLM group (*P* < 0.05). **(D–J)** Relative mRNA expression levels of *Artn, Aurka, Depdc1, Sdc1, PfkM, Mertk,* and *Fbp2*, as measured by qRT-PCR. All genes were significantly upregulated in the BLM group compared to the PBS group (*P* < 0.05), n = 12 of each group and data are represented as the means ± SEM, Mann-Whitney U test.

In order to further verify the results of cell experiments *in vitro* and simulate the pathological process of patients with pulmonary fibrosis as much as possible, we successfully constructed a bleomycin-induced pulmonary fibrosis mouse model for *in-vivo* experiments. The results of HE, Masson staining and Western blot showed that compared with the control group, the bleomycin group had significantly increased lung tissue inflammation and collagen deposition (Figures 12A, B). And Body weight changes showed that compared with the control group, the bleomycin group manifested a significant weight loss (Figure 12C). RT-qPCR results indicated that the glycolysis-related genes *Artn, Aurka, Sdc1, Mertk*, and *Fbp2* were significantly upregulated in bleomycin-induced mouse lung tissue, whereas *Pfkm* was significantly downregulated. These findings are in agreement with our previous datasets, suggesting that these six differentially expressed glycolysis-related genes may play an important role in the development of pulmonary fibrosis. In contrast, the expression level of *Depdc1* was markedly elevated compared to the earlier datasets, which may be attributable to differences in the timing or grouping criteria for bleomycin intervention in the animal models (Figures 12D–J).

## Discussion

In this study, we analyzed the relationship between IPF and GRGs using the GEO dataset. Our initial analysis identified 14 potential marker genes (*PFKM, PKLR, FBP2, AGRN, ARTN, AURKA, CXCR4, DEPDC1, MERTK, PLOD2, SDC1, TPBG, VCAN,* and *PGP*). ROC analysis demonstrated that these genes had diagnostic potential for IPF. To further investigate the prognostic significance of these genes, we incorporated survival data from IPF patients and constructed a survival model using LASSO regression. This analysis revealed that seven of the genes (*PFKM, FBP2, ARTN, AURKA, DEPDC1, MERTK*, and *SDC1*) exhibited predictive value for IPF prognosis. These results highlight the prognostic relevance of various GRGs in IPF. The selection of these seven genes was a pragmatic approach aimed at improving the efficiency and interpretability of the model, while ensuring the retention of essential prognostic information. Further investigation through immunoreactivity and single-cell sequencing analyses revealed a association between GRGs and immune cell infiltration in the IPF microenvironment. Notably, *DEPDC1, AURKA*, and *MERTK* displayed the highest expression levels in macrophages, among the four major cell types analyzed. To verify these findings, we constructed a bleomycin-induced pulmonary fibrosis mouse model. In line with our predictions, the expression of the seven prognostic genes was significantly increased in fibrotic lung tissue, reinforcing the potential link between glycolysis-related gene expression and pulmonary fibrosis. These findings provide a foundation for further exploring the role of glycolysis in the progression of pulmonary fibrosis.

Metabolic reprogramming, specifically the shift towards glycolysis, has emerged as a critical driver of fibrotic processes, facilitating the activation of fibroblasts and the deposition of ECM (Baudo et al., 2023; Yan et al., 2023) Previous studies have shown that key glycolytic enzymes such as *PFKM* and *PKM2* are upregulated in fibrotic tissues and contribute to myofibroblast differentiation, a hallmark of fibrosis (Zhang et al., 2023; Xie et al., 2015; Gao et al., 2022)^.^ Our findings support this, as we observed a significant differential expression in *PFKM* expression in IPF tissues, suggesting a crucial role of glycolysis in the fibrotic process. Moreover, genes like *ARTN* and *FBP2*, identified in our study, have also been implicated in regulating fibroblast activity and ECM production, reinforcing the idea that glycolysis not only meets energy demands but also drives fibroblast activation and fibrosis progression (Sontake et al., 2017; Ding et al., 2017; Kasam et al., 2020).These findings align with the work of Xie et al., who showed that glycolytic enzymes are upregulated in lung fibroblasts during the fibrotic process, suggesting that glycolysis promotes fibroblast activation through both metabolic and signaling pathways (Yan et al., 2023; Xie et al., 2015)^.^

It is well-established that immune cell infiltration and activation are pivotal in driving the inflammatory and fibrotic responses observed in IPF (Tiwari et al., 2024; Nuovo et al., 2012; Yang et al., 2009). A growing body of evidence suggests that glycolysis is central to the regulation of immune cell function. For instance, Tina Tylek et al. demonstrated that glycolysis supports macrophage polarization towards a pro-inflammatory and pro-fibrotic phenotype, which exacerbates ECM remodeling and fibrosis (Tylek et al., 2024; Cheng et al., 2020; Widiapradja et al., 2024). Similarly, studies by Xia et al. have highlighted that glycolysis promotes the formation of neutrophil extracellular traps (NETs), contributing to tissue damage and fibrosis in inflammatory diseases (Xia et al., 2024; Wu and Yang, 2024). Our study provides further support for this link, as we observed that the glycolysis-related genes *DEPDC1*, *AURKA,* and *MERTK* were highly expressed in macrophages within the IPF microenvironment. This suggests that these genes may modulate macrophage activation and function through glycolytic pathways, contributing to fibrosis. The role of macrophages in IPF is particularly relevant, as they are key players in the initiation and progression of fibrosis (Zhang et al., 2018; Ge et al., 2024; Qin et al., 2022). Macrophages not only promote inflammation but also stimulate the fibrotic response through the secretion of profibrotic cytokines and the regulation of fibroblast function (Jeny et al., 2021; Wynn and Barron, 2010). Recent studies have demonstrated that glycolytic reprogramming enhances macrophage activation and the release of pro-inflammatory cytokines, contributing to the overall fibrotic process. Our findings suggest that targeting these glycolysis-related genes may offer a novel strategy for modulating macrophage activity in IPF.

In addition to their role in immune modulation and fibroblast activation, glycolysis-related genes directly contribute to the fibrotic microenvironment by regulating ECM remodeling and cellular interactions (Ezhilarasan, 2021; Willette et al., 2021; Wei et al., 2024). Genes like *VCAN* and *PLOD2* are involved in ECM regulation and have been implicated in fibrotic diseases. *VCAN*, for example, is known to promote ECM deposition and fibroblast activation (Kasam et al., 2020; Harten et al., 2021). In our study, we observed that several glycolysis-related genes, including *VCAN*, were upregulated in IPF tissues, indicating that these genes might contribute to ECM remodeling through metabolic and signaling pathways. This is consistent with the previous findings of Jeremy Herrera et al., who showed that ECM components like versican and collagen are regulated by metabolic pathways, including glycolysis, in the context of fibrosis (Brotman et al., 1988; Wight, 2017; Herrera et al., 2018). The significance of the ECM in fibrosis is extremely important. The ECM not only provides structural support to tissues but also regulates cell behavior through interactions with integrins and other cell surface receptors (Watson et al., 2016; Ford and Rajagopalan, 2018; Hudson et al., 2017). By influencing ECM production and turnover, glycolysis-related genes may play a crucial role in regulating tissue homeostasis and fibrosis. Our identification of these genes in the context of IPF further highlights their potential as key regulators of the fibrotic microenvironment.

In conclusion, this study highlights the role of glycolysis-related genes in IPF, suggesting potential biomarkers and therapeutic targets. However, several limitations exist. First, reliance on GEO datasets and animal models may not fully reflect human IPF complexity. Although associations between glycolysis-related genes and macrophage activation were observed, the exact molecular mechanisms remain unclear. Future research should focus on elucidating how glycolytic reprogramming affects macrophage polarization and ECM production. Clinical trials are also needed to evaluate the therapeutic potential of targeting these genes in IPF, offering insights into the feasibility of metabolic-based treatments.

## Conclusion

In summary, our study is important in understanding glycolysis’s role and the interaction between glycolysis and immune function in IPF. Additionally, GRDEGs might be a potential target for future IPF diagnosis and treatment.

## Data Availability

The original contributions presented in the study are included in the article/Supplementary Material, further inquiries can be directed to the corresponding authors.
