# Supplementary material for: Identification of glycolysis-related gene signatures for prognosis and therapeutic targeting in idiopathic pulmonary fibrosis
Source: Front Pharmacol. 2025 Feb 28;16:1486357. doi: 10.3389/fphar.2025.1486357 (PMC11906445; doi:10.3389/fphar.2025.1486357)
Supplement: Supplementary file 1 [file Table1.docx]

Supplementary Table 1 Comprehensive Summary of GEO Datasets Analyzed

| GEO Dataset ID | Data Type | Sample Source | Sample Groups | Number of Samples per Group |
| --- | --- | --- | --- | --- |
| GSE70866 | Bulk RNA-seq | Bronchoalveolar lavage (BAL) fluid cells | IPF, Normal | IPF: 176; Normal: 20 |
| GSE218997 | Bulk RNA-seq | Lung tissue from male C57 mice | Bleomycin (BLM)-induced fibrosis, Control | BLM: 59; Control: 78 |
| GSE128033 | Single-cell RNA-seq | Lung tissue collected during transplant surgeries | IPF | IPF: 8 |
