# Supplementary material for: Identification of glycolysis-related gene signatures for prognosis and therapeutic targeting in idiopathic pulmonary fibrosis
Source: Front Pharmacol. 2025 Feb 28;16:1486357. doi: 10.3389/fphar.2025.1486357 (PMC11906445; doi:10.3389/fphar.2025.1486357)
Supplement: Supplementary file 2 [file Table2.docx]

Supplementary Table 2 Glycolysis-Related Gene Sets and Their Descriptions in the MSigDB Database.

| Name | Genes | Description |
| --- | --- | --- |
| BIOCARTA_GLYCOLYSIS_PATHWAY | 3 | Glycolysis Pathway |
| KEGG_GLYCOLYSIS_GLUCONEOGENESIS | 62 | Glycolysis / Gluconeogenesis |
| MODULE_306 | 26 | Glycolysis and TCA cycle |
| REACTOME_GLYCOLYSIS | 74 | Glycolysis |
| HALLMARK_GLYCOLYSIS | 200 | Genes encoding proteins involved in glycolysis and gluconeogenesis. |
| WP_GLYCOLYSIS_IN_SENESCENCE | 11 | Glycolysis in senescence |
| WP_GLYCOLYSIS_AND_GLUCONEOGENESIS | 45 | Glycolysis and gluconeogenesis |
| WP_AEROBIC_GLYCOLYSIS | 12 | Aerobic glycolysis |
