# Supplementary material for: Identification of glycolysis-related gene signatures for prognosis and therapeutic targeting in idiopathic pulmonary fibrosis
Source: Front Pharmacol. 2025 Feb 28;16:1486357. doi: 10.3389/fphar.2025.1486357 (PMC11906445; doi:10.3389/fphar.2025.1486357)
Supplement: Supplementary file 4 [file Table3.docx]

Supplementary Table 3 The mouse primer sequences used in the study.

| **Gene** | Forward | Reverse |
| --- | --- | --- |
| AURKA | 5’-CTGGATGCTGCAAACGGATAG-3’ | 5′- CGAAGGGAACAGTGGTCTTAACA -3′ |
| Depdc1 | 5′- TTGGTTGTTTGTGGCTACATCA′ | 5′- GCTGCATTTTCTTGGCACATC -3′ |
| SDC1 | 5′- ACTTCACCTTTGAAACATCTGGG -3′ | 5′- CATCCGGTACAGCATGAAAGC -3′ |
| ARTN | 5′- CGAGCTGATACGTTTCCGCTT -3′ | 5′- AGACGGCCTCATAGCGAGT -3′ |
| MERTK | 5′- CCTAACCGTACCTGGTCTGAC -3′ | 5′- GGGAGGGGATTACTTTGATGTTG -3′ |
| FBP2 | 5′- TGGCCTCCATCGGAACTATATT -3′ | 5′- TGGCCTCCATCGGAACTATATT -3′ |
| PFKM | 5′- GCGACTTGCTGAATGATCTCC -3′ | 5′- CATTGTCGATTGAGCCAACCA -3′ |
